# Vaccination of hamsters with *Opisthorchis viverrini* extracellular vesicles and vesicle-derived recombinant tetraspanins induces antibodies that block vesicle uptake by cholangiocytes and reduce parasite burden after challenge infection

**DOI:** 10.1371/journal.pntd.0007450

**Published:** 2019-05-28

**Authors:** Sujittra Chaiyadet, Javier Sotillo, Watchara Krueajampa, Sophita Thongsen, Paul J. Brindley, Banchob Sripa, Alex Loukas, Thewarach Laha

**Affiliations:** 1 Department of Parasitology, Faculty of Medicine, Khon Kaen University, Khon Kaen, Thailand; 2 Centre for Molecular Therapeutics, Australian Institute of Tropical Health and Medicine, James Cook University, Cairns, Australia; 3 Department of Microbiology, Immunology and Tropical Medicine, and Research Center for Neglected Diseases of Poverty, School of Medicine & Health Sciences, George Washington University, Washington, DC, United States of America; 4 Department of Pathology, Faculty of Medicine, Khon Kaen University, Khon Kaen, Thailand; IRNASA, CSIC, SPAIN

## Abstract

**Background:**

The liver fluke *Opisthorchis viverrini* infects several million people in Southeast Asia. Adult flukes live in the bile ducts of humans, where they cause hepatobiliary pathology, including cholangiocarcinoma. Here, we investigated the potential of extracellular vesicles (EVs) secreted by the fluke and defined recombinant proteins derived from EVs to generate protective immunity in a hamster vaccination-challenge model.

**Methodology/Principal findings:**

EVs isolated from the excretory-secretory products of *O*. *viverrini* and two recombinant EV surface proteins encoding the large extracellular loops (LEL) of *Ov*-TSP-2 (r*Ov*-TSP-2) and *Ov*-TSP-3 (r*Ov*-TSP-3) were adjuvanted and used to vaccinate hamsters intraperitoneally followed by challenge infection with *O*. *viverrini* metacercariae. The number of adult flukes recovered from hamsters immunized with EVs, r*Ov*-TSP-2, r*Ov*-TSP-3 and r*Ov*-TSP-2+r*Ov*-TSP-3 were significantly reduced compared to control animals vaccinated with adjuvant alone. The number of eggs per gram feces was also significantly reduced in hamsters vaccinated with r*Ov*-TSP-2 compared to controls, but no significant differences were found in the other groups. The average length of worms recovered from hamsters vaccinated with EVs, r*Ov*-TSP-2 and r*Ov*-TSP-3 was significantly shorter than that of worms recovered from the control group. Anti-EV IgG levels in serum and bile were significantly higher in hamsters vaccinated with EVs compared to control hamsters both pre- and post-challenge. In addition, levels of anti-r*Ov*-TSP antibodies in the serum and bile were significantly higher than control hamsters both pre- and post-challenge. Finally, antibodies against r*Ov*-TSP-2 and r*Ov*-TSP-3 blocked uptake of EVs by human primary cholangiocyte *in vitro*, providing a plausible mechanism by which these vaccines exert partial efficacy and reduce the intensity of *O*. *viverrini* infection.

**Conclusion/Significance:**

Liver fluke EVs and recombinant tetraspanins derived from the EV surface when administered to hamsters induce antibody responses that block EV uptake by target bile duct cells and exert partial efficacy and against *O*. *viverrini* challenge.

## Author summary

Cholangiocarcinoma (CCA) is a significant public health problem in countries throughout Southeast Asia. In these areas CCA has a strong association with chronic infection with the food-borne liver fluke *Opisthorchis viverrini*. Current control of the infection relies on chemotherapy and health education, however these approaches are not sustainable in isolation. Hence, there is an urgent need for a vaccine against this neglected tropical disease. A vaccine against *O*. *viverrini* would confer anti-cancer protection in similar fashion to the acclaimed vaccine for human papillomavirus and cervical cancer. Toward this goal, secreted extracellular vesicles (EVs) of *O*. *viverrini* and recombinant proteins from the surface of EVs were generated and tested as vaccines in a hamster challenge model. Vaccination of hamsters with EVs and recombinant proteins induced production of antibodies in serum and bile, and those antibodies blocked uptake of EVs by primary bile duct cells *in vitro*. Challenge of vaccinated hamsters with infective stage flukes markedly reduced adult fluke recovery compared to the adjuvant control group. This is the first report of successful vaccination of hamsters with *O*. *viverrini* EVs and recombinant vesicle surface proteins, and provides proof-of-concept for development of subunit vaccines for this carcinogenic infection.

## Introduction

The human liver fluke *Opisthorchis viverrini* is endemic in different countries of Southeast Asia including Thailand, Lao PDR, Cambodia, southern part of Vietnam and Myanmar [1, 2]. Furthermore, liver fluke infection is associated with a high incidence of liver pathology including cholangiocarcinoma (CCA) [3, 4]. Current control efforts rely on drug treatment and health education; however, they are not sustainable [5, 6]. Hence, it is essential to develop new interventions for long-term protection against *O*. *viverrini* infection, and a vaccine approach is an attractive strategy to reduce parasite burden and achieve ultimate eradication of the parasite.

*O*. *viverrini*-induced hepatobiliary damage is multi-factorial, and includes several factors such as mechanical damage of the epithelium by the suckers of the worm, secreted parasite metabolites [7, 8] and different immunopathological processes [9]. The metabolic products of *O*. *viverrini* are highly immunogenic and stimulate immunopathology [8], and include tegumental and secreted proteins comprising more than 300 proteins [10]. In addition to secretion of soluble protein, we have previously reported that *O*. *viverrini* secretes exosome-like extracellular vesicles (*Ov*-EVs) [11]. EVs are 40–100 nm in size and of endocytic origin; they are enriched in various lipids, nucleic acids and proteins including tetraspanins, heat shock protein and actin [12]. *O*. *viverrini* tetraspanins (*Ov*-TSPs), belonging to both the CD9 and CD63 families, were found to be enriched in *Ov*-EVs and abundantly expressed on the outermost tegument of adult worms [13, 14]. *Ov*-TSPs play an important role in dynamic host-parasite interactions, particularly maintenance of tegument membrane integrity, and their suppression using RNA interference results in a vacuolated tegument *in vitro* [13, 14]. EVs have been used as protective vaccines in mouse models of intestinal helminths, including the nematodes, *Trichuris muris* [15] and *Heligmosomoides polygyrus* [16] and the trematode, *Echinostoma caproni* [17]. Given the abundance of tetraspanins on the surface of fluke EVs [18, 19] and the ability of antibodies against *Ov*-TSP-1 to block uptake of EVs by host cells [13], we propose that fluke TSPs could be used as vaccines to interrupt host-parasite communication [20]. Moreover, TSPs from the surface of schistosomes have proven to be efficacious vaccines in animal models of blood fluke infection [21].

In the present study we assessed the vaccine potential of *O*. *viverrini* EVs and recombinant *Ov*-TSPs derived from the EVs surface and the ability of antibodies from vaccinated animals to block EVs uptake by host cells.

## Materials and methods

### Preparation of *O*. *viverrini* metacercariae

*O*. *viverrini* metacercariae were prepared as previously described [22]. Briefly, cyprinid fishes from natural sources were homogenized with a blender and the homogenized fish was added to pepsin solution (0.25% pepsin powder, 15% HCl in normal saline solution—NSS) at a ratio of 1:3, followed by incubation at 37°C for 1 hour to enable digestion. The digested solution was filtered through 1,000, 300, and 106 μm meshes. The debris obtained by filtering with the 106 μm mesh was washed and repeatedly sedimented with NSS until clear. Sediments were examined for metacercariae under a dissecting microscope. *O*. *viverrini* metacercariae were collected and stored in sterile NSS at 4°C until used.

### Production of recombinant *Ov*-TSP-2 and -3 and rabbit antisera

The large extracellular loop (LEL) from *Ov*-TSP-2 and *Ov*-TSP-3 were recombinantly produced as fusions with thioredoxin (TRX) using the plasmid pET32a+ (Novagen, USA) in *Escherichia coli* and purified as previously described [19]. New Zealand rabbits were immunized with r*Ov*-TSP-2 or -3 and antisera was collected as previously described [19].

### Isolation of *O*. *viverrini* extracellular vesicles (*Ov*-EVs)

EVs were isolated from the excretory/secretory (ES) products released by adult *O*. *viverrini* (*Ov*ES) as described previously with modifications [11, 23, 24]. Briefly, *O*. *viverrini* adult worms were cultured in RPMI-1640 containing 1% glucose, antibiotics (Penicillin-Streptomycin 100 μg, Invitrogen, USA) and the 1 μM protease inhibitor E64 (Thermo Scientific, USA). Worms were maintained *in vitro* at 37°C and supernatants containing the *Ov*ES were collected twice each day for up to 7 days and centrifuged at 2,090 *g* for 10 min to remove the eggs. *Ov*ES was concentrated and buffer exchanged to 1x PBS using a 10 kDa cut-off purification column (Amicon, Merk Millipore, USA). One (1) ml of concentrated *Ov*ES was centrifuged at 500 *g*, 2,000 *g*, 4,000 *g* and 12,000 *g* for 30 min each to remove cell debris. The supernatant was filtered using a 0.22 μm filter (Sartorius, Germany), and subsequently pelleted by ultracentrifugation at 110,000 *g* for 3 hours. The OptiPrep density gradient ultracentrifugation (ODG) was prepared by diluting a 60% Iodixanol solution (Sigma Aldrich, USA) with 0.25 M sucrose in 10 mM Tris-HCl, pH 7.2 to make 40%, 20%, 10% and 5% iodixanol solutions and then 1.0 ml of these solutions was layered in decreasing density in an ultracentrifuge tube. Pelleted *O*. *viverrini* EVs (*Ov*-EVs) were layered on top of the gradient and further centrifuged at 110,000 *g* for 18 h at 4°C. The fractions with a density of 1.12–1.24 g/mL were pooled and buffer exchanged to PBS using 100 kDa cut-off purification columns (Amicon, Merk Millipore, USA) and resuspended in 200 μl of PBS.

### Vaccination and challenge of hamsters

Male Syrian golden hamsters 6–8 weeks-old were used for vaccination studies. Sample size was calculated using two sample inference for comparing two means (https://www.stat.ubc.ca/~rollin/stats/ssize/n2.html) [25]. A sample size of n = 9 was selected on the assumption of a vaccine trial resulting in a mean of 40 worms per hamster for μ1 (adjuvant control) and 25 worms per hamster for μ2 (test group), σ = 11, two-sided test, with α = 0.05 and power of 0.80. A total of 45 hamsters were divided into 5 groups (9 hamsters/group) including 1) vaccinated with 10 μg of *Ov*-EVs, 2) vaccinated with 50 μg of r*Ov*-LEL-TSP-2, 3) vaccinated with 50 μg of r*Ov*-LEL-TSP-3, 4) vaccinated with 50 μg of r*Ov*-LEL-TSP-2 plus 50 μg of r*Ov*-LEL-TSP-3. Both EVs and recombinant proteins were formulated with an equal volume of a colloidal suspension of aluminium hydroxide gel (alum) (Invivogen, USA) and CpG ODN 1826 (10 μg) (Invivogen, USA). A fifth group of hamsters were injected with 10 μg of alum/CpG only (adjuvant control group) as a control group. Vaccines were injected intraperitoneally on days 0, 14 and 28; and hamsters were challenged with 50 metacercariae via the oral route on day 42 (2 weeks after last vaccination). Hamsters were finally sacrificed at 8 weeks post-challenge. Blood was collected for serum by heart puncture after euthanasia and bile was collected from the gall bladder. Whole livers were collected to investigate the number of flukes.

### Faecal egg counts and worm recovery

Faeces from individual hamsters from each group were collected 6 weeks after challenge, and eggs per gram feces (EPG) were counted and calculated using a modified formalin-ether acetate technique [26]. Briefly, 2 grams of hamster feces were fixed in 10 ml of 10% formalin solution, filtered through two layers of gauze, the filtered solution centrifuged at 500 g for 2 minutes, and the supernatant discarded. The pellet was re-suspended with 7 ml of 10% formalin, vigorously mixed with 3 ml ethyl acetate and then centrifuged at 500 *g* for 5 minutes. The pellet was dissolved in 1 ml of 10% formalin solution and examined in duplicate under light microscopy at 400x magnification. EPG was calculated as follows: (average of number eggs x total drops of fecal solution)/ g of feces. Whole livers from the hamsters were dissected in NSS and adult worms were collected and counted. The percent reduction of worm recovery was calculated as previously described [27]: % Worm reduction = ((Wc–We) / Wc) x 100 (Wc = worm burden in control group, We = worm burden in experiment group). To measure worm length, 21.4–42.2% of the total worms recovered from each group were randomly selected. Worms were washed three times with NSS and fixed in pre-warmed 10% formalin. Worms were photographed under microscopy and worm length was measured using NIS-Element software (Nikon, Japan).

### Indirect ELISA for specific IgG

Specific IgG against r*Ov*-TSP-2, r*Ov*-TSP-*3*, *Ov*ES, or *Ov*-EVs was measured in the serum and bile by indirect ELISA. Hamster serum and bile were collected as described above. Polystyrene flat bottom96-well ELISA plates (NUNC-F96, Fisher Scientific, USA) were coated with 100 μl of r*Ov*-TSP-2 or r*Ov*-TSP-3 or *Ov*ES (1 μg/ml) or *Ov*-EVs (2 μg/ml) overnight at 4°C in coating buffer (0.05 M Na_2_CO_3_/NaHCO_3_, pH 9.6). Plates were washed with PBS 0.05% Tween-20 (PBST) and, then, blocked with 200 μl 5% skim milk in PBST for 2 hours at 37°C. One hundred (100) μl of sera (1:2,000 in PBST/2% skim milk) or bile (1:200 in PBST/2% skim milk) were added and incubated for 2 hours at 37°C. The plates were washed with PBST and probed with 100 μl of anti-hamster IgG conjugate HRP (BioRad, USA) (1:2000 in PBST for serum and 1:1,000 for bile). After washing with PBST, the plates were developed by adding 50 μl TMB (Thermo Fisher Scientific, USA) for 20 minutes, and reaction was stopped by adding 50 μl of 2N H_2_SO_4_. The colorimetric reaction was read at a wavelength of 450 nm on a Spectra Max microplate reader (Molecular Devices, USA).

### Western blot

Five (5) μg of *Ov*ES, *Ov*EVs, *Ov*ES depleted of *Ov*EVs, r*Ov*-TSP-2 and r*Ov*-TSP3 were loaded and electrophoresed in a 15% SDS-PAGE gel and proteins were transferred to a nitrocellulose membrane (Mini Trans-Blot Cell, Bio-Rad, USA). Membranes were probed with vaccinated hamster serum (1:200) by incubation at 4°C overnight followed by incubation with anti-hamster IgG-HRP (1:1,000) at 25°C for 2 h. The reaction color was developed by adding Luminata Forte Western HRP Substrate (Millipore, USA).

### Blocking of *O*. *viverrini* EVs internalization by host cells

Five (5) μg of *Ov*-EVs were labeled with PKH67 (Sigma-aldrich, USA) following the manufacturer’s instructions and incubated with anti-*Ov*-TSP-2 or -TSP-3 rabbit sera [19], with vaccinated hamster sera (*Ov*-TSP-2, *Ov*-TSP-3, *Ov*-TSP-2 plus -3 and *Ov*-EVs) or with control sera (adjuvant alone) at a 1:2.5 dilution for 1 hour at room temperature. *Ov*-EVs-antibody complexes were washed by 1x PBS using 100 kDa cut-off purification columns (Amicon, Merk Millipore, USA) and cultured with H69 cells for 2 hours. Nuclei were stained with 2 μg/ml of Hoechst (Invitrogen, USA) for 15 minutes. The image was taken using a Carl Zeiss confocal microscope (LSM800, USA) at 200x of the original magnification. Thirty cells from 2 biological replicates were analyzed for fluorescence intensity using imageJ version 1.50i.

### Statistical analysis

All data are represented as mean ± SD of three independent experiments using Graphpad Prism Software Version 6.03 (www.graphpad.com). Data from worm burden, EPG, worm length, ELISA experiments and fluorescence intensity were evaluated by Student’s t-test.

### Ethics statement

Male Syrian (golden) hamsters (*Mesocricetus auratas*) were reared at the animal facilities of the Faculty of Medicine, Khon Kaen University. Experimentation protocols were approved by the Animal Ethics Committee of Khon Kaen University according to the Ethics of Animal Experimentation of the National Research Council of Thailand, with the approval number ACUC KKU 10/2559.

## Results

### *O*. *viverrini* tetraspanin antibodies inhibit internalization of *Ov*-EVs by human cholangiocytes

**Fig 1:**
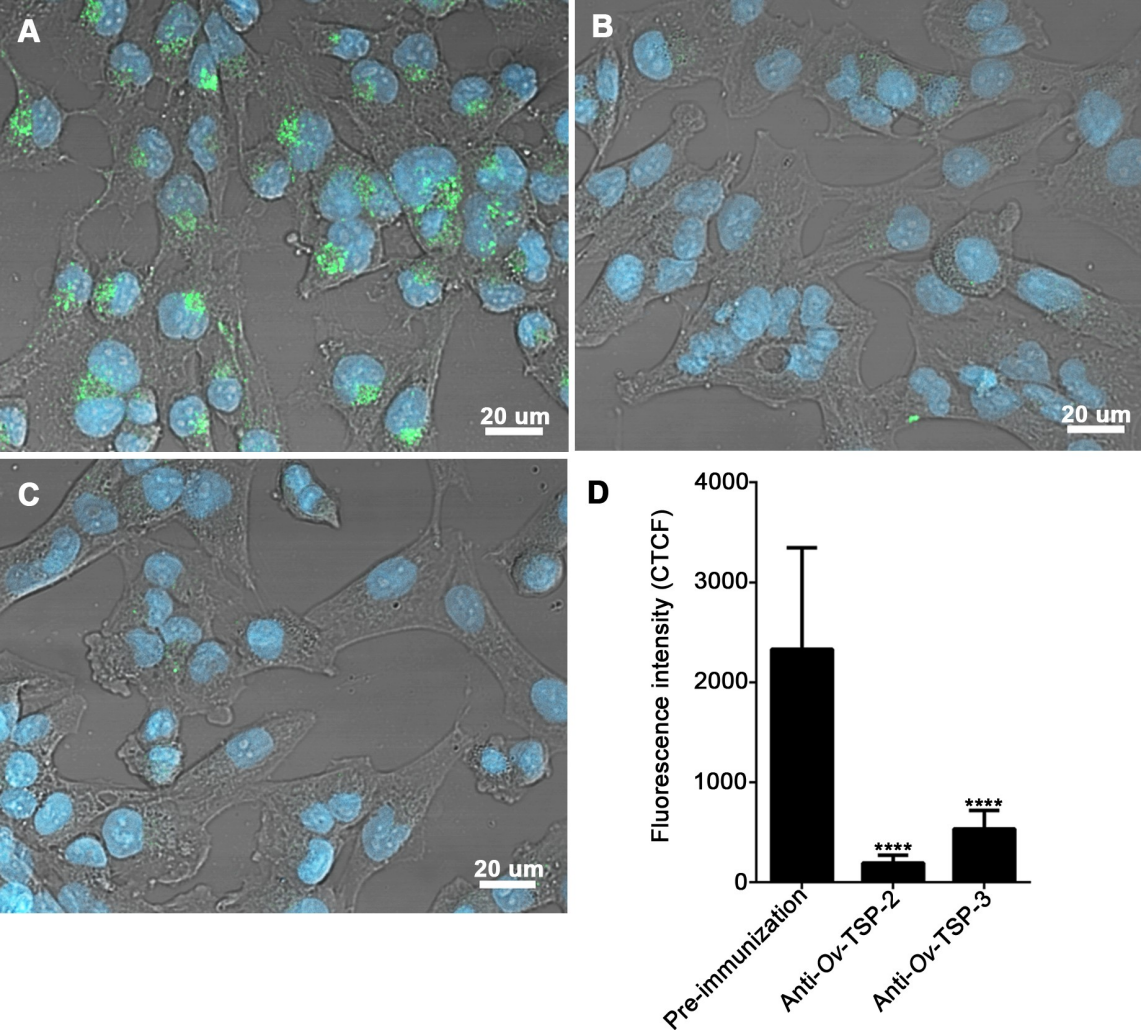
*Ov*-TSP antisera blocks *O*. *viverrini* EVs internalization by human cholangiocytes. PKH67-labeled *Ov*-EVs were incubated with rabbit antisera to r*Ov*-TSP-2 or r*Ov*-TSP-3 or pre-immunization serum prior to co-culture with H69 cholangiocytes. Panel A = rabbit pre-immunization serum, panel B = rabbit antiserum to r*Ov*-TSP-2 1:2.5, panel C = rabbit antiserum to r*Ov*-TSP-3 1:2.5. Panel D shows fluorescence intensity at 490 nm (green channel). The nuclei were stained by Hoechst (blue color) (**** *P*<0.0001).

H69 human cholangiocytes actively internalize *Ov*-EVs [11]. Co-culture of fluorescently labeled *Ov*-EVs with antisera from rabbits vaccinated with r*Ov*-TSP-2 and r*Ov*-TSP-3 resulted in 91% (****P<0.0001) and 77% (****P<0.0001) reductions respectively in uptake of EVs by cells compared to control cells that were cultured with pre-immunization rabbit serum (Fig 1).

### IgG levels in the serum and bile of hamsters vaccinated with *Ov*-EVs and r*Ov*-TSPs

**Fig 2:**
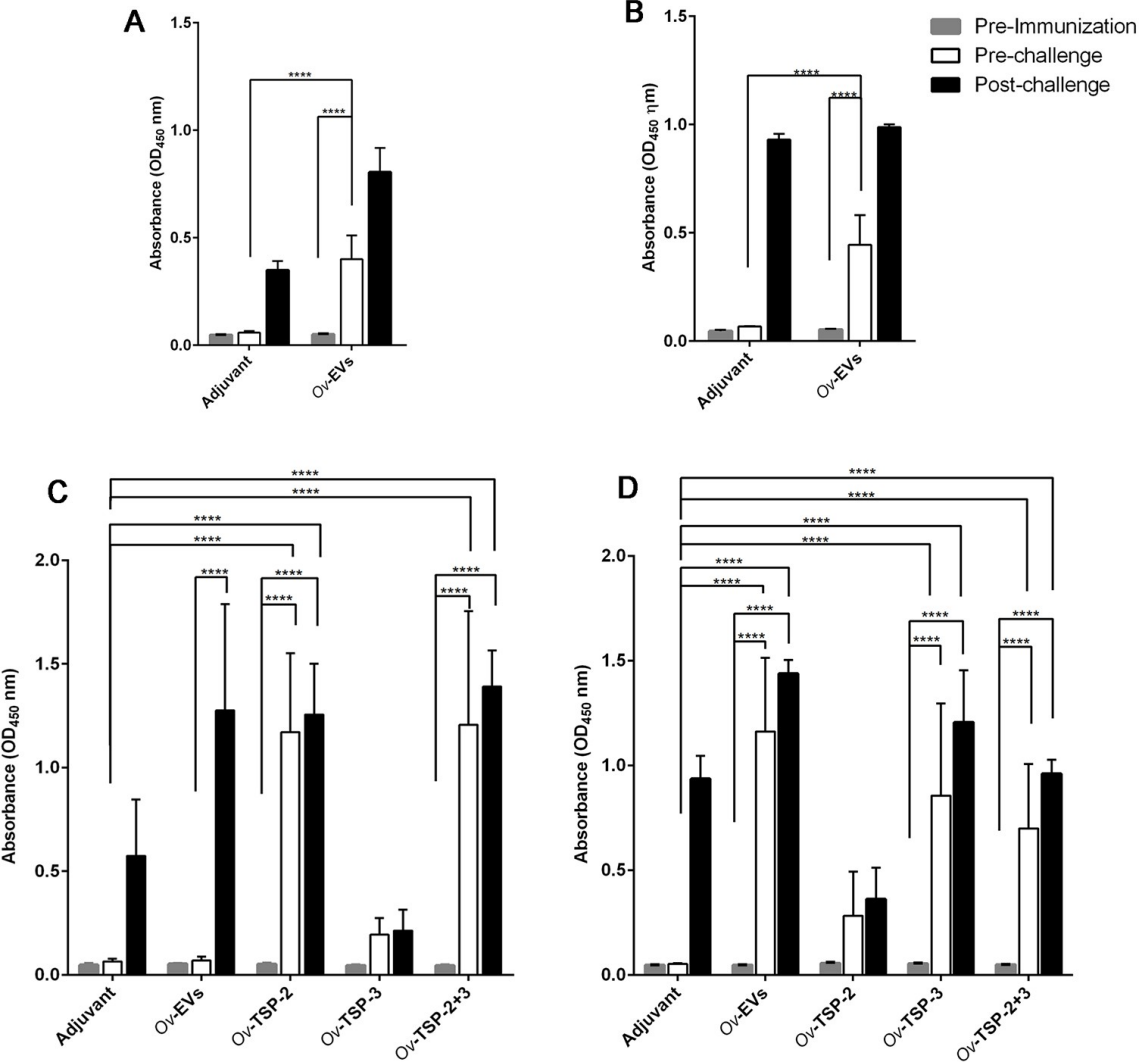
Serum IgG levels in vaccinated hamsters. Serum IgG was measured from hamsters immunized with *Ov*-EVs, r*Ov-*TSP-2, r*Ov*-TSP-3 and r*Ov*-TSP-2+r*Ov*-TSP-3 at pre-immunization, pre-challenge and post-challenge. The IgG levels were measured against *Ov*ES (A), *Ov*-EVs (B), r*Ov*-TSP-2 (C) and r*Ov*-TSP-3 (D). Results represent the mean absorbance measured at 450 nm for each group. Significant differences are denoted by an asterisk over the bar (**** *P*<0.0001).

Hamsters vaccinated with *Ov*-EVs showed a significant increase in serum IgG against *Ov*ES (Fig 2A) and *Ov*-EVs (Fig 2B) when compared with pre-immunization sera and sera from the adjuvant control group (*P*<0.0001). On the other hand, the IgG levels against *Ov*ES post-vaccination but pre-challenge in hamsters vaccinated with r*Ov*-TSP-2, r*Ov*-TSP-3 and r*Ov*-TSP-2+r*Ov*-TSP-3 were not statistically significant compared to the adjuvant control group (S1 Fig).

The total serum IgG against r*Ov*-TSP-2 in hamsters immunized with r*Ov*-TSP-2 and r*Ov*-TSP-2+r*Ov*-TSP-3 was significantly higher at pre-challenge and post-challenge compared to pre-immunization and adjuvant control sera (*P*<0.0001) (Fig 2C).

Likewise, the serum IgG against r*Ov*-TSP-3 levels in hamsters immunized with *Ov*-EVs, r*Ov*-TSP-3 or r*Ov*-TSP-2+r*Ov*-TSP-3 were significantly higher pre- and post-challenge than pre-immunization and adjuvant control sera (*P*<0.0001) (Fig 2D).

**Fig 3:**
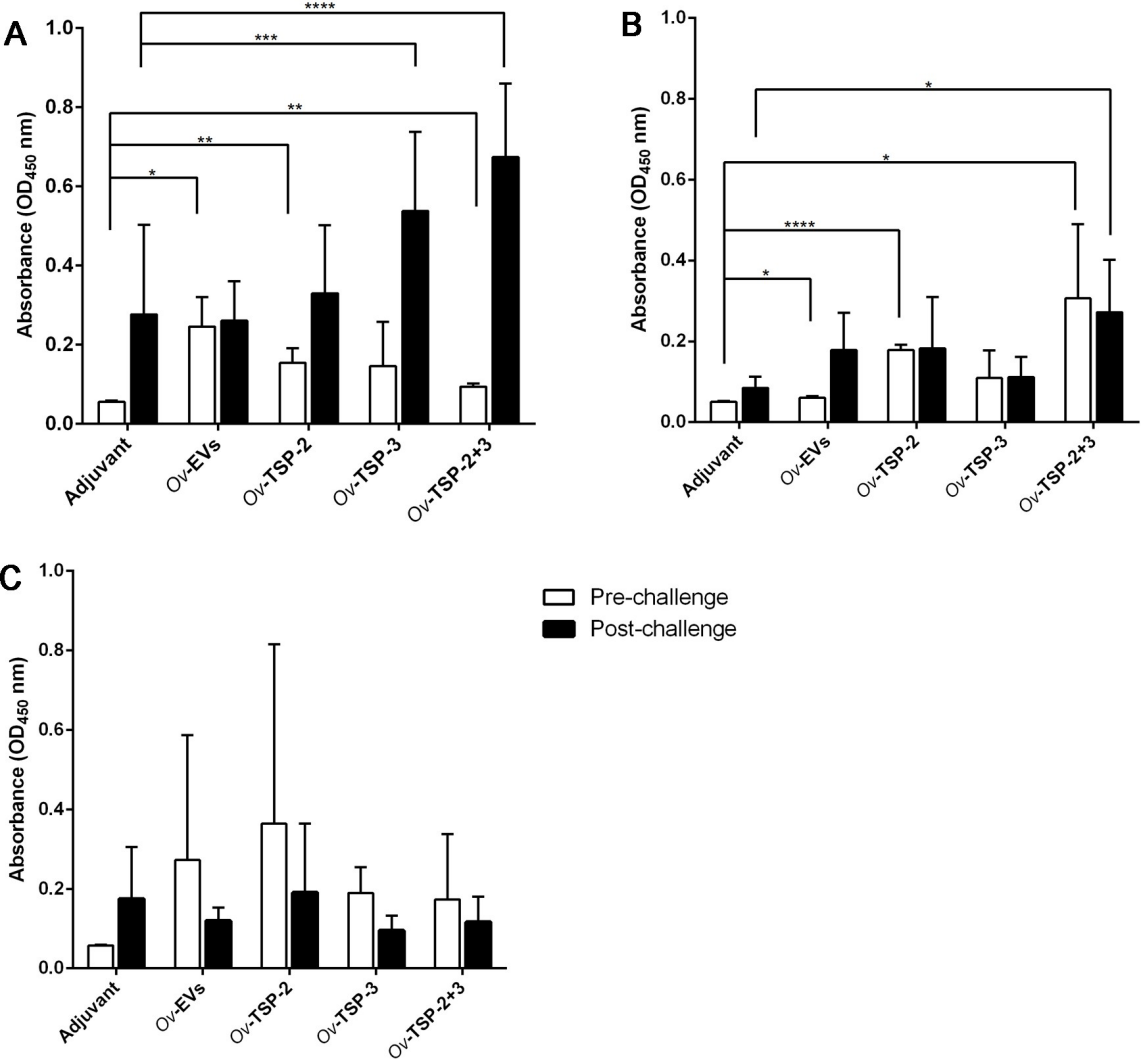
IgG levels in bile of vaccinated hamsters. Bile IgG was measured by ELISA at pre-challenge and post-challenge against *Ov*ES (A), r*Ov*-TSP-2 (B) and r*Ov*-TSP-3 (C). Results represent the mean absorbance measured at 450 nm for each group. Significant differences are denoted by an asterisk over the bar (**** *P*<0.0001, *** *P*<0.001, ** *P*<0.01, * *P*<0.05).

Total bile IgG against *Ov*ES was significantly higher post-vaccination but pre-challenge in hamsters vaccinated with *Ov*-EVs (*P*< 0.05), r*Ov*-TSP-2 (*P*<0.01) and r*Ov*-TSP-2+r*Ov*-TSP-3 (*P*<0.01) when compared with adjuvant control bile, while IgG levels in post-challenge samples were significantly higher in r*Ov*-TSP-2 (*P*<0.001) and r*Ov*-TSP-2+r*Ov*-TSP-3 (*P*<0.0001) vaccinated groups than in the adjuvant group (Fig 3A).

IgG levels against r*Ov*-TSP-2 in the bile of hamsters immunized with r*Ov*-TSP-2 and r*Ov*-TSP-2+r*Ov*-TSP-3 were significantly higher than bile IgG from the adjuvant control group (Fig 3B). Furthermore, the IgG levels in the bile of r*Ov*-TSP-2+r*Ov*-TSP-3 vaccinated animals were significantly (*P*<0.05) higher at pre-challenge and post-challenge compared to the same samples from the adjuvant group. In the *Ov*-EVs vaccinated group, bile IgG levels against r*Ov*-TSP-2 post-challenge were higher than those obtained pre-challenge (*P*<0.05) and in the adjuvant control groups, but these differences did not reach significance (Fig 3B).

IgG antibody levels in bile against r*Ov*-TSP-3 were not statistically different in the pre-challenge or post-challenge samples in any group when compared to the adjuvant group (Fig 3C).

### Vaccination with *Ov*-EVs and *Ov*-TSPs decreased worm burden in hamsters challenged with *O*. *viverrini*

**Fig 4:**
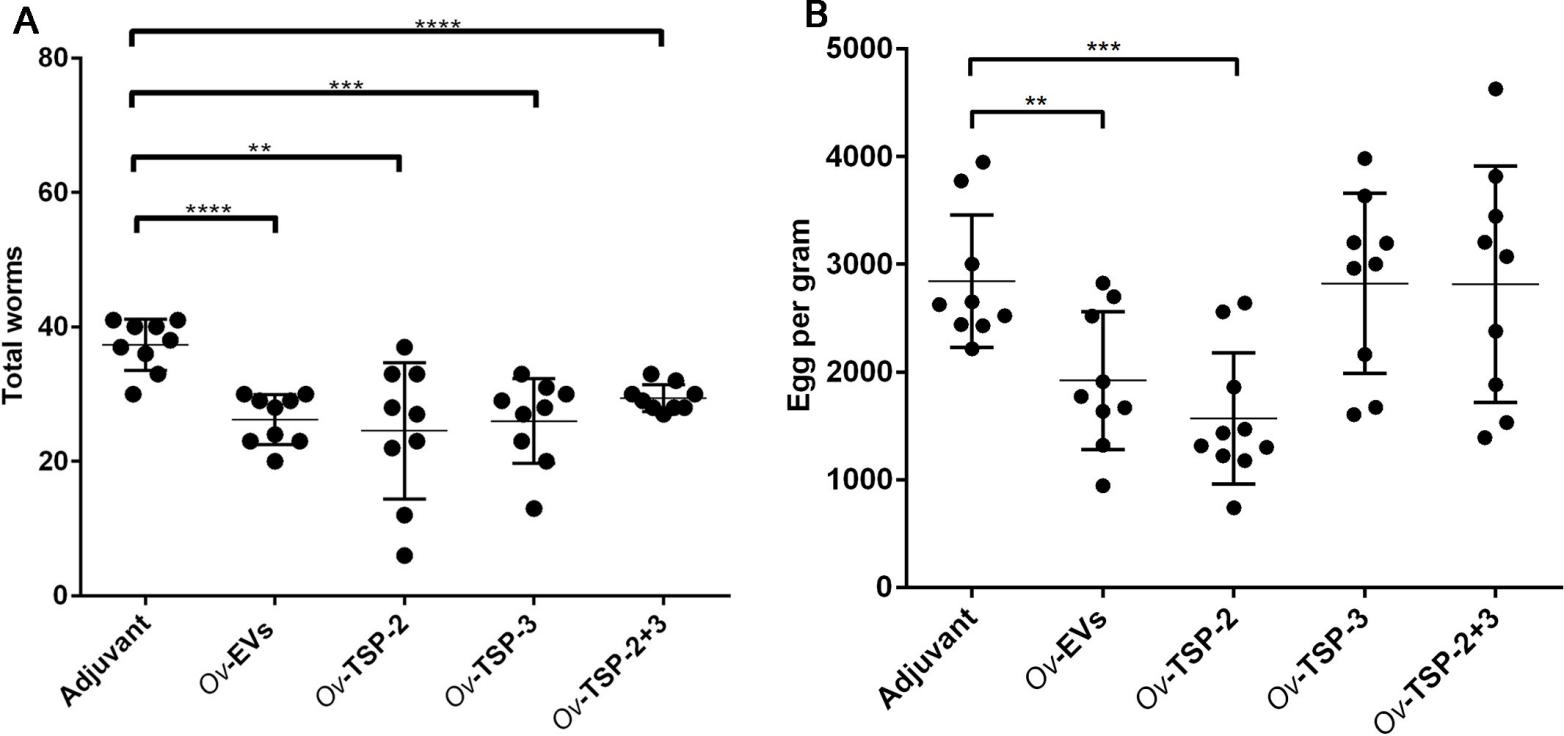
Vaccinated hamsters have significantly fewer worms than control animals. Panel A, Worm burden from each hamster and worm reduction rate in hamsters vaccinated with *O*. *viverrini* EVs (*Ov*-EVs), r*Ov*-TSP-2, r*Ov*-TSP-3 and r*Ov*-TSP-2+r*Ov*-TSP-3 and control alum/CpG (adjuvant). ** *P*<0.01, *** *P*<0.001, **** *P*<0.0001. Panel B = Number of eggs per gram of feces (EPG) from each vaccinated hamster with mean and SD bar shown (** *P*<0.01, *** *P*<0.001).

Hamsters immunized with *Ov*-EVs, r*Ov*-TSP-2, r*Ov*-TSP-3 and r*Ov*-TSP-2+r*Ov*-TSP-3 had significantly lower worm burdens than the control group post-challenge (*P*<0.05) (Fig 4A). The number of worms was reduced by 27% (*P*<0.05), 34% (*P*<0.01), 30% (*P*<0.001) and 21% (*P*<0.001) respectively when compared with the adjuvant control group (Fig 4A). Furthermore, mean EPG in hamsters vaccinated with *Ov*-EVs and r*Ov*-TSP-2 were reduced by 32% (*P*<0.01) and 41% (*P*<0.001) respectively compared to the adjuvant control group (Fig 4B). Vaccination with r*Ov*-TSP-3 and r*Ov*-TSP-2+r*Ov*-TSP-3 had no effect on EPG when compared with the control group (Fig 4B).

### Growth retardation of worms recovered from hamsters vaccinated with *Ov*-EVs and r*Ov*-TSPs

**Fig 5:**
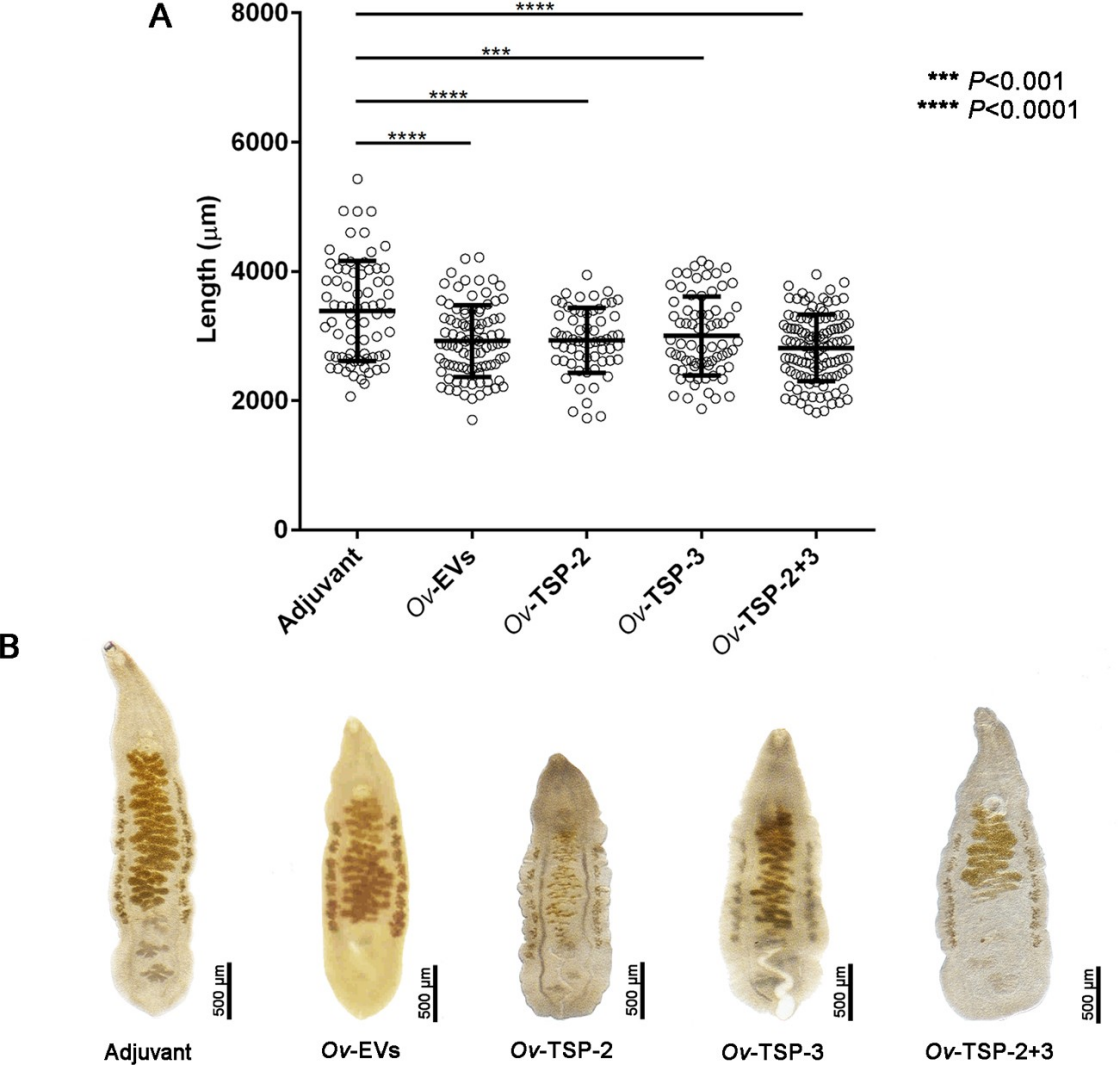
Significant stunting is evident in worms recovered from vaccinated animals. The body length of 21.4–42.2% of randomly selected worms recovered from each experimental group of vaccinated hamsters was measured using the NIS-element program (A). Flukes from vaccinated animals were significantly shorter than those from hamsters that received adjuvant alone (B).

The body length of recovered worms from hamsters vaccinated with *Ov*-EVs, r*Ov*-TSP-2, r*Ov*-TSP-3 and r*Ov*-TSP-2+r*Ov*-TSP-3 was significantly (*P*<0.001) shorter than worms recovered from the control adjuvant group (Fig 5A). The average length of examined worms from hamsters vaccinated with *Ov*-EVs, r*Ov*-TSP-2, r*Ov*-TSP-3 and r*Ov*-TSP-2+r*Ov*-TSP-3 was 2.92±0.55, 2.93±0.50, 3.01±0.61, 2.82 ±0.51 mm, respectively while the average length of worms from control adjuvant group was 33.89±0.77 mm. A representation of the morphology of the worms from each group is shown in Fig 5B.

### Sera from vaccinated hamsters recognize *O*. *viverrini* proteins

*Ov*ES, *Ov*ES depleted of EVs (dEVs), r*Ov-*TSP-2 and r*Ov*-TSP-3 were probed with a pooled serum from each group of vaccinated hamsters by western blot. *Ov*ES was only detected by pre-challenge serum from the *Ov*-EVs vaccinated group. The r*Ov*-TSP-2 protein was detected by pooled sera from pre-challenge hamsters vaccinated with r*Ov*-TSP-2, r*Ov*-TSP-3 and r*Ov*-TSP-2+r*Ov*-TSP-3, while the r*Ov*-TSP-3 protein was recognized by pooled sera from hamsters vaccinated with *Ov*-EVs, r*Ov*-TSP-2, r*Ov*-TSP-3 and r*Ov*-TSP-2+r*Ov*-TSP-3. As expected, none of the samples were detected by pooled serum from hamsters vaccinated with adjuvant alone (S2A Fig). Post-challenge sera from all vaccinated groups reacted against all samples (*Ov*ES, r*Ov*-TSP-2, r*Ov*-TSP-3, dEVs and *Ov*-EVs), indicating that the native extracts (*Ov*ES and EVs) as well as recombinant TSP-2 and TSP-3 are immunogenic in the course of a natural infection (S2B Fig).

### Antibodies of vaccinated hamsters block uptake of *Ov*-EVs by host cells

**Fig 6:**
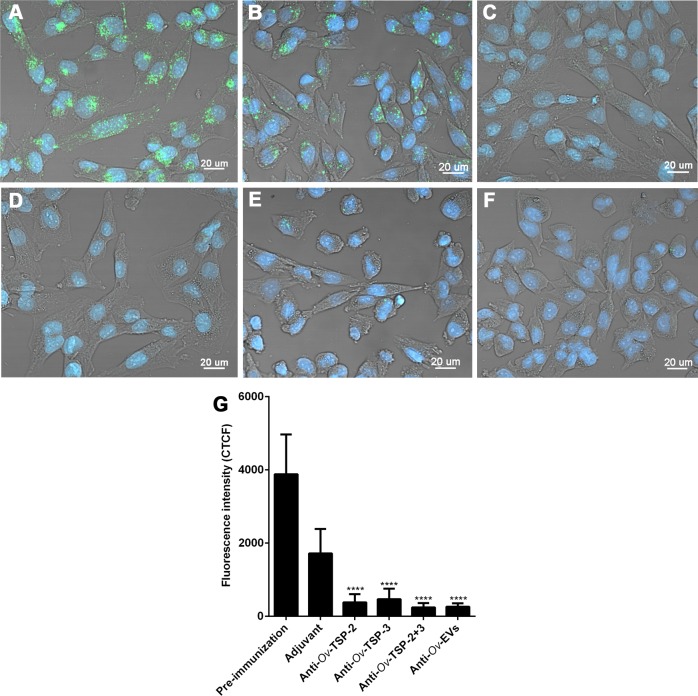
Sera from hamsters vaccinated with r*Ov*-TSPs block *O*. *viverrini* EV internalization by host cholangiocytes. PKH67 labeled *Ov*-EVs was incubated with hamster antisera to r*Ov*-TSP-2 or r*Ov*-TSP-3 or r*Ov*-TSP-2+r*Ov*-TSP-3 or adjuvant or pre-immunization prior to co-culture with H69 cholangiocytes. Panel A = hamster pre-immunized serum, panel B = Adjuvant hamster serum, panel C = hamster antiserum to r*Ov*-TSP-2, panel D = hamster antiserum to r*Ov*-TSP-3, panel E = hamster antiserum to r*Ov*-TSP-2 plus -3 and panel F = hamster antiserum to *Ov*-EVs. Panel G shows fluorescence intensity of internalization at 490 nm (green channel). The nuclei were stained by Hoechst (blue color) (**** *P*<0.0001).

PKH67-labelled *Ov*-EVs were incubated with pooled sera of hamsters vaccinated with r*Ov*-TSP-2, r*Ov*-TSP-3, r*Ov*-TSP-2+r*Ov*-TSP-3, *Ov*-EVs and from the control groups at a dilution of 1:2.5 before being cultured with H69 cholangiocytes. *Ov*-EVs uptake was significantly reduced more than 77% and 72% after incubation with r*Ov*-TSP-2 and r*Ov*-TSP -3 vaccinated hamster serum, respectively (Fig 6C, 6D and 6G) when compared with pre-immunization serum and serum from the adjuvant group (Fig 6A). Likewise, antisera to r*Ov*-TSP-2+r*Ov*-TSP-3 and antisera to *Ov*-EVs blocked *Ov*-EVs internalization by cholangicytes by more than 80% (Fig 6E, 6F and 6G).

## Discussion

The presence of EVs in *Ov*ES products precipitated the hypothesis that these vesicles are important in host-parasite communication and immunopathogenesis [11]. EVs have also been characterized in others trematodes such as *Echinostoma caproni* [28], *Fasciola hepatica* [29] and *Schistosoma spp*. [18, 30, 31]. Moreover, other helminths such as the gastrointestinal nematodes *H*. *polygyrus* [16, 32] and *N*. *brasiliensis* [33] also secrete EVs enriched in specific proteins and miRNAs and can suppress the immunological response *in vivo*.

In the present study we have shown that immunization of hamsters with *Ov*-EVs reduced both worms burden and egg production, and that IgG antibody levels specific to *Ov*-EVs were significantly raised in serum and in bile of vaccinated hamsters. The development of the adult parasites was significantly hampered in hamsters vaccinated with *Ov*-EVs, as demonstrated by stunted parasite growth in vaccinated hamsters. Other groups have also studied the ability of EVs to control trematode and other helminth infections. For instance, purified EVs from *Echinostoma caproni* used to vaccinate mice prior to parasite challenge resulted in elevated antibody (IgG) responses but vaccination did not reduce the number of adult worms in the gut after challenge infection [17]. Vaccination of mice with EVs from the nematode *H*. *polygyrus* resulted in worm reduction of 82% [16].

The use of native helminth EVs as vaccines is not feasible due to the difficulty in culturing sufficient numbers of worms to collect the required amount of EVs material, however the utility of sera from EVs-vaccinated animals as a discovery tool is evident. With that in mind, we reasoned that proteins that are abundant on the surface of *Ov*-EVs might impart protective immunity by blocking fluke EVs-host cell interactions. To our knowledge, this is the first report of vaccination with recombinant proteins specifically aimed at inducing antibodies against EVs surface proteins and subsequent blocking of EVs uptake by host cells.

*Ov*-TSP-2 and *Ov*-TSP-3 are members of the CD63 family of tetraspanins and have been shown to be abundantly expressed in the *O*. *viverrini* tegument as well as enriched in *Ov*-EVs [11]. Vaccination of hamsters with r*Ov*-TSP-2 and r*Ov*-TSP-3 strong IgG responses that were readily detectable in both sera and bile–the fluid surrounding adult flukes in the biliary tract. The presence of specific IgG in the bile might be a result of diffusion from the blood vessels into the gall bladder where they impact on resident flukes. Previous vaccine studies have used crude *O*. *viverrini* antigens to immunize hamsters prior to challenge with metacercariae [6], but to our knowledge this is the first report of successful vaccination of hamsters with *O*. *viverrini* recombinant antigens. The utility of tetraspanins as helminth vaccines is evident in schistosomiasis where *Sm*-TSP-2 is one of the leading vaccine candidates for a human schistosomiasis vaccine [21] and has recently completed phase 1 clinical trials [34].

Antibodies against proteins specific to the *Ov*-EVs membrane such as the TSPs from sera from vaccinated animals (pre-challenge) significantly blocked EVs internalization by cholangiocytes. These antibodies might block the ability of the parasite to communicate with its host, thereby impairing the establishment and growth of the parasite by interrupting its ability to suppress inflammation. Worms recovered from vaccinated animals were stunted, supporting this hypothesis. It has been previously shown that blocking *Ov*-EVs internalization decreases the levels of IL-6 (a cytokine heavily implicated in the development of fibrosis) and cell proliferation [11]. We suggest that antibodies specific to the *Ov*-EVs surface may be performing neutralizing roles by blocking the ability of fluke EVs to hijack host cholangiocytes and interfere with induction of wound healing processes that repair damaged biliary tissue [35, 36] and promote long-term survival of the flukes. Clearly, other molecular mechanisms that are independent of EVs are utilized by liver flukes to communicate with their hosts, including proteins, peptides, glycans and lipids that direct interact with host cells [37].

In contrast to our findings reported herein where antibodies to EV surface TSPs block uptake of vesicles by cholangiocytes, a recent report described enhanced uptake by macrophages of *F*. *hepatica* EVs in the presence of antibodies against a CD63-like TSP on the vesicle surface [38]. This is likely due to opsonisation of EVs and FcR-mediated uptake by antigen presenting cells [16, 38] as opposed to distinct EV uptake by non-phagocytic target cells such as cholangiocytes that we describe herein.

In summary, immunization with r*Ov*-TSP-2 and *Ov*-EVs elicit robust antibody responses which is key for protection against fluke infection, resulting in reduction of worms and egg burden. Moreover, antibodies produced against r*Ov*-TSP-2 significantly blocked internalization of *Ov*-EVs by cholangiocytes. The tetraspanin proteins present in the membrane of EVs are key in cell contact and uptake [39], and impairing this interaction could be blocking important host-parasite interactions affecting the survival of the parasite. This is the first study to determine the vaccine efficacy of *Ov*-EVs or the recombinant tetraspanins r*Ov*-TSP-2 and r*Ov*-TSP-3 against a challenge infection with metacercariae of *O*. *viverrini* in hamsters, and it supports the notion that targeting interactions between fluke EVs and their mammalian host cells holds promise for the discovery of novel vaccine candidates.

## Supporting information

S1 Fig Serum IgG against *Ov*-ES of vaccinated hamsters. ELISA showing IgG levels against *Ov*-ES of pre-immunized, pre-challenge or post-challenge hamster sera from each vaccinated group.
(TIF)

S2 Fig Western blot analysis of the recognition of *O. viverrini* proteins by sera from vaccinated hamsters. *Ov*ES, r*Ov*-TSP-2, r*Ov*-TSP-3, *Ov-*ES-depleted EVs (dEVs) and *Ov*-EVs were probed with pre-challenge hamster sera (A) or post-challenge hamster sera from each vaccination group (B).
(TIF)

## References

[1] Sithithaworn P, Andrews RH, Nguyen VD, Wongsaroj T, Sinuon M, Odermatt P, et al The current status of opisthorchiasis and clonorchiasis in the Mekong Basin. Parasitology international. 2012;61(1):10–6. Epub 2011/09/07. 10.1016/j.parint.2011.08.014 21893213 PMC3836690

[2] Aung WPP, Htoon TT, Tin HH, Thinn KK, Sanpool O, Jongthawin J, et al First report and molecular identification of Opisthorchis viverrini infection in human communities from Lower Myanmar. PloS one. 2017;12(5):e0177130 Epub 2017/05/05. 10.1371/journal.pone.0177130 28472153 PMC5417708

[3] Sriamporn S, Pisani P, Pipitgool V, Suwanrungruang K, Kamsa-ard S, Parkin DM. Prevalence of Opisthorchis viverrini infection and incidence of cholangiocarcinoma in Khon Kaen, Northeast Thailand. Tropical medicine & international health: TM & IH. 2004;9(5):588–94. Epub 2004/05/01. 10.1111/j.1365-3156.2004.01234.x . 15117303

[4] Khuntikeo N, Titapun A, Loilome W, Yongvanit P, Thinkhamrop B, Chamadol N, et al Current Perspectives on Opisthorchiasis Control and Cholangiocarcinoma Detection in Southeast Asia. Frontiers in medicine. 2018;5:117 Epub 2018/05/17. 10.3389/fmed.2018.00117 29765958 PMC5938629

[5] Soukhathammavong P, Odermatt P, Sayasone S, Vonghachack Y, Vounatsou P, Hatz C, et al Efficacy and safety of mefloquine, artesunate, mefloquine-artesunate, tribendimidine, and praziquantel in patients with *Opisthorchis viverrini*: a randomised, exploratory, open-label, phase 2 trial. Lancet Infect Dis. 2011;11(2):110–8. Epub 2010/11/30. 10.1016/S1473-3099(10)70250-4 . 21111681

[6] van den Enden E. Pharmacotherapy of helminth infection. Expert Opin Pharmacother. 2009;10(3):435–51. Epub 2009/02/05. 10.1517/14656560902722463 . 19191680

[7] Harinasuta T, Riganti M, Bunnag D. *Opisthorchis viverrini* infection: pathogenesis and clinical features. Arzneimittelforschung. 1984;34(9B):1167–9. Epub 1984/01/01. . 6542384

[8] Sripa B, Brindley PJ, Mulvenna J, Laha T, Smout MJ, Mairiang E, et al The tumorigenic liver fluke *Opisthorchis viverrini*—multiple pathways to cancer. Trends Parasitol. 2012;28(10):395–407. Epub 2012/09/06. 10.1016/j.pt.2012.07.006 22947297 PMC3682777

[9] Haswell-Elkins MR, Sithithaworn P, Mairiang E, Elkins DB, Wongratanacheewin S, Kaewkes S, et al Immune responsiveness and parasite-specific antibody levels in human hepatobiliary disease associated with *Opisthorchis viverrini* infection. Clin Exp Immunol. 1991;84(2):213–8. Epub 1991/05/01. 10.1111/j.1365-2249.1991.tb08151.x 2025950 PMC1535388

[10] Mulvenna J, Sripa B, Brindley PJ, Gorman J, Jones MK, Colgrave ML, et al The secreted and surface proteomes of the adult stage of the carcinogenic human liver fluke *Opisthorchis viverrini*. Proteomics. 2010;10(5):1063–78. Epub 2010/01/06. 10.1002/pmic.200900393 20049860 PMC3038172

[11] Chaiyadet S, Sotillo J, Smout M, Cantacessi C, Jones MK, Johnson MS, et al Carcinogenic Liver Fluke Secretes Extracellular Vesicles That Promote Cholangiocytes to Adopt a Tumorigenic Phenotype. J Infect Dis. 2015;212(10):1636–45. Epub 2015/05/20. 10.1093/infdis/jiv291 25985904 PMC4621255

[12] Raposo G, Stoorvogel W. Extracellular vesicles: exosomes, microvesicles, and friends. J Cell Biol. 2013;200(4):373–83. Epub 2013/02/20. 10.1083/jcb.201211138 23420871 PMC3575529

[13] Chaiyadet S, Smout M, Laha T, Sripa B, Loukas A, Sotillo J. Proteomic characterization of the internalization of *Opisthorchis viverrini* excretory/secretory products in human cells. Parasitol Int. 2017;66(4):494–502. Epub 2016/02/14. 10.1016/j.parint.2016.02.001 26873540 PMC5149449

[14] Piratae S, Tesana S, Jones MK, Brindley PJ, Loukas A, Lovas E, et al Molecular characterization of a tetraspanin from the human liver fluke, *Opisthorchis viverrini*. PLoS neglected tropical diseases. 2012;6(12):e1939 Epub 2012/12/14. 10.1371/journal.pntd.0001939 23236532 PMC3516575

[15] Shears RK, Bancroft AJ, Hughes GW, Grencis RK, Thornton DJ. Extracellular vesicles induce protective immunity against *Trichuris muris*. Parasite immunology. 2018;40(7):e12536 Epub 2018/05/11. 10.1111/pim.12536 29746004 PMC6055854

[16] Coakley G, McCaskill JL, Borger JG, Simbari F, Robertson E, Millar M, et al Extracellular Vesicles from a Helminth Parasite Suppress Macrophage Activation and Constitute an Effective Vaccine for Protective Immunity. Cell reports. 2017;19(8):1545–57. Epub 2017/05/26. 10.1016/j.celrep.2017.05.001 28538175 PMC5457486

[17] Trelis M, Galiano A, Bolado A, Toledo R, Marcilla A, Bernal D. Subcutaneous injection of exosomes reduces symptom severity and mortality induced by *Echinostoma caproni* infection in BALB/c mice. International journal for parasitology. 2016;46(12):799–808. Epub 2016/10/25. 10.1016/j.ijpara.2016.07.003 . 27590846

[18] Sotillo J, Pearson M, Potriquet J, Becker L, Pickering D, Mulvenna J, et al Extracellular vesicles secreted by *Schistosoma mansoni* contain protein vaccine candidates. International journal for parasitology. 2016;46(1):1–5. Epub 2015/10/16. 10.1016/j.ijpara.2015.09.002 . 26460238

[19] Chaiyadet S, Krueajampa W, Hipkaeo W, Plosan Y, Piratae S, Sotillo J, et al Suppression of mRNAs encoding CD63 family tetraspanins from the carcinogenic liver fluke *Opisthorchis viverrini* results in distinct tegument phenotypes. Scientific reports. 2017;7(1):14342 Epub 2017/11/01. 10.1038/s41598-017-13527-5 29084967 PMC5662742

[20] Mekonnen GG, Pearson M, Loukas A, Sotillo J. Extracellular vesicles from parasitic helminths and their potential utility as vaccines. Expert review of vaccines. 2018;17(3):197–205. Epub 2018/01/23. 10.1080/14760584.2018.1431125 . 29353519

[21] Tran MH, Pearson MS, Bethony JM, Smyth DJ, Jones MK, Duke M, et al Tetraspanins on the surface of *Schistosoma mansoni* are protective antigens against schistosomiasis. Nat Med. 2006;12(7):835–40. Epub 2006/06/20. 10.1038/nm1430 . 16783371

[22] Laha T, Pinlaor P, Mulvenna J, Sripa B, Sripa M, Smout MJ, et al Gene discovery for the carcinogenic human liver fluke, *Opisthorchis viverrini*. BMC Genomics. 2007;8:189 Epub 2007/06/26. 10.1186/1471-2164-8-189 17587442 PMC1913519

[23] Kalra H, Adda CG, Liem M, Ang CS, Mechler A, Simpson RJ, et al Comparative proteomics evaluation of plasma exosome isolation techniques and assessment of the stability of exosomes in normal human blood plasma. Proteomics. 2013;13(22):3354–64. Epub 2013/10/12. 10.1002/pmic.201300282 . 24115447

[24] Chaiyadet S, Smout M, Johnson M, Whitchurch C, Turnbull L, Kaewkes S, et al Excretory/secretory products of the carcinogenic liver fluke are endocytosed by human cholangiocytes and drive cell proliferation and IL6 production. International journal for parasitology. 2015;45(12):773–81. Epub 2015/07/19. 10.1016/j.ijpara.2015.06.001 26187786 PMC4912216

[25] Rosner B. Hypothesis Testing: Categorical Data/Estimation of Sample Size and Power for Comparing Two Binomial Proportions Fundamentals of biostatistics. 7 ed Boston: Brooks/Cole, Cengage Learning; 2011.

[26] Elkins DB, Haswell-Elkins M, Anderson RM. The epidemiology and control of intestinal helminths in the Pulicat Lake region of Southern India. I. Study design and pre- and post-treatment observations on *Ascaris lumbricoides* infection. Trans R Soc Trop Med Hyg. 1986;80(5):774–92. Epub 1986/01/01. 10.1016/0035-9203(86)90384-6 . 3603617

[27] Jittimanee J, Sermswan RW, Kaewraemruaen C, Barta JR, Macinnes JI, Maleewong W, et al Protective immunization of hamsters against *Opisthorchis viverrini* infection is associated with the reduction of TGF-beta expression. Acta tropica. 2012;122(2):189–95. Epub 2012/01/24. 10.1016/j.actatropica.2012.01.010 . 22266215

[28] Marcilla A, Trelis M, Cortes A, Sotillo J, Cantalapiedra F, Minguez MT, et al Extracellular vesicles from parasitic helminths contain specific excretory/secretory proteins and are internalized in intestinal host cells. PLoS One. 2012;7(9):e45974 Epub 2012/10/03. 10.1371/journal.pone.0045974 23029346 PMC3454434

[29] Cwiklinski K, de la Torre-Escudero E, Trelis M, Bernal D, Dufresne PJ, Brennan GP, et al The Extracellular Vesicles of the Helminth Pathogen, *Fasciola hepatica*: Biogenesis Pathways and Cargo Molecules Involved in Parasite Pathogenesis. Mol Cell Proteomics. 2015;14(12):3258–73. Epub 2015/10/22. 10.1074/mcp.M115.053934 26486420 PMC4762619

[30] Zhu L, Liu J, Dao J, Lu K, Li H, Gu H, et al Molecular characterization of *S*. *japonicum* exosome-like vesicles reveals their regulatory roles in parasite-host interactions. Scientific reports. 2016;6:25885 Epub 2016/05/14. 10.1038/srep25885 27172881 PMC4865838

[31] Nowacki FC, Swain MT, Klychnikov OI, Niazi U, Ivens A, Quintana JF, et al Protein and small non-coding RNA-enriched extracellular vesicles are released by the pathogenic blood fluke *Schistosoma mansoni*. Journal of extracellular vesicles. 2015;4:28665 Epub 2015/10/08. 10.3402/jev.v4.28665 26443722 PMC4595467

[32] Buck AH, Coakley G, Simbari F, McSorley HJ, Quintana JF, Le Bihan T, et al Exosomes secreted by nematode parasites transfer small RNAs to mammalian cells and modulate innate immunity. Nat Commun. 2014;5:5488 Epub 2014/11/26. 10.1038/ncomms6488 25421927 PMC4263141

[33] Eichenberger RM, Ryan S, Jones L, Buitrago G, Polster R, Montes de Oca M, et al Hookworm Secreted Extracellular Vesicles Interact With Host Cells and Prevent Inducible Colitis in Mice. Frontiers in immunology. 2018;9:850 Epub 2018/05/16. 10.3389/fimmu.2018.00850 29760697 PMC5936971

[34] Merrifield M, Hotez PJ, Beaumier CM, Gillespie P, Strych U, Hayward T, et al Advancing a vaccine to prevent human schistosomiasis. Vaccine. 2016;34(26):2988–91. Epub 2016/04/03. 10.1016/j.vaccine.2016.03.079 . 27036511

[35] Smout MJ, Laha T, Mulvenna J, Sripa B, Suttiprapa S, Jones A, et al A granulin-like growth factor secreted by the carcinogenic liver fluke, Opisthorchis viverrini, promotes proliferation of host cells. PLoS pathogens. 2009;5(10):e1000611 Epub 2009/10/10. 10.1371/journal.ppat.1000611 19816559 PMC2749447

[36] Smout MJ, Sotillo J, Laha T, Papatpremsiri A, Rinaldi G, Pimenta RN, et al Carcinogenic Parasite Secretes Growth Factor That Accelerates Wound Healing and Potentially Promotes Neoplasia. PLoS pathogens. 2015;11(10):e1005209 Epub 2015/10/21. 10.1371/journal.ppat.1005209 26485648 PMC4618121

[37] Suttiprapa S, Sotillo J, Smout M, Suyapoh W, Chaiyadet S, Tripathi T, et al Opisthorchis viverrini Proteome and Host-Parasite Interactions. Advances in parasitology. 2018;102:45–72. Epub 2018/11/18. 10.1016/bs.apar.2018.06.002 . 30442310 PMC12212976

[38] de la Torre-Escudero E, Gerlach JQ, Bennett APS, Cwiklinski K, Jewhurst HL, Huson KM, et al Surface molecules of extracellular vesicles secreted by the helminth pathogen Fasciola hepatica direct their internalisation by host cells. PLoS neglected tropical diseases. 2019;13(1):e0007087 Epub 2019/01/19. 10.1371/journal.pntd.0007087 30657764 PMC6355031

[39] Andreu Z, Yanez-Mo M. Tetraspanins in extracellular vesicle formation and function. Frontiers in immunology. 2014;5:442 Epub 2014/10/04. 10.3389/fimmu.2014.00442 25278937 PMC4165315

