# Supplementary figures and images for: Vaccination of hamsters with Opisthorchis viverrini extracellular vesicles and vesicle-derived recombinant tetraspanins induces antibodies that block vesicle uptake by cholangiocytes and reduce parasite burden after challenge infection
Source: PLoS Negl Trop Dis. 2019 May 28;13(5):e0007450. doi: 10.1371/journal.pntd.0007450 (PMC6555531; doi:10.1371/journal.pntd.0007450)

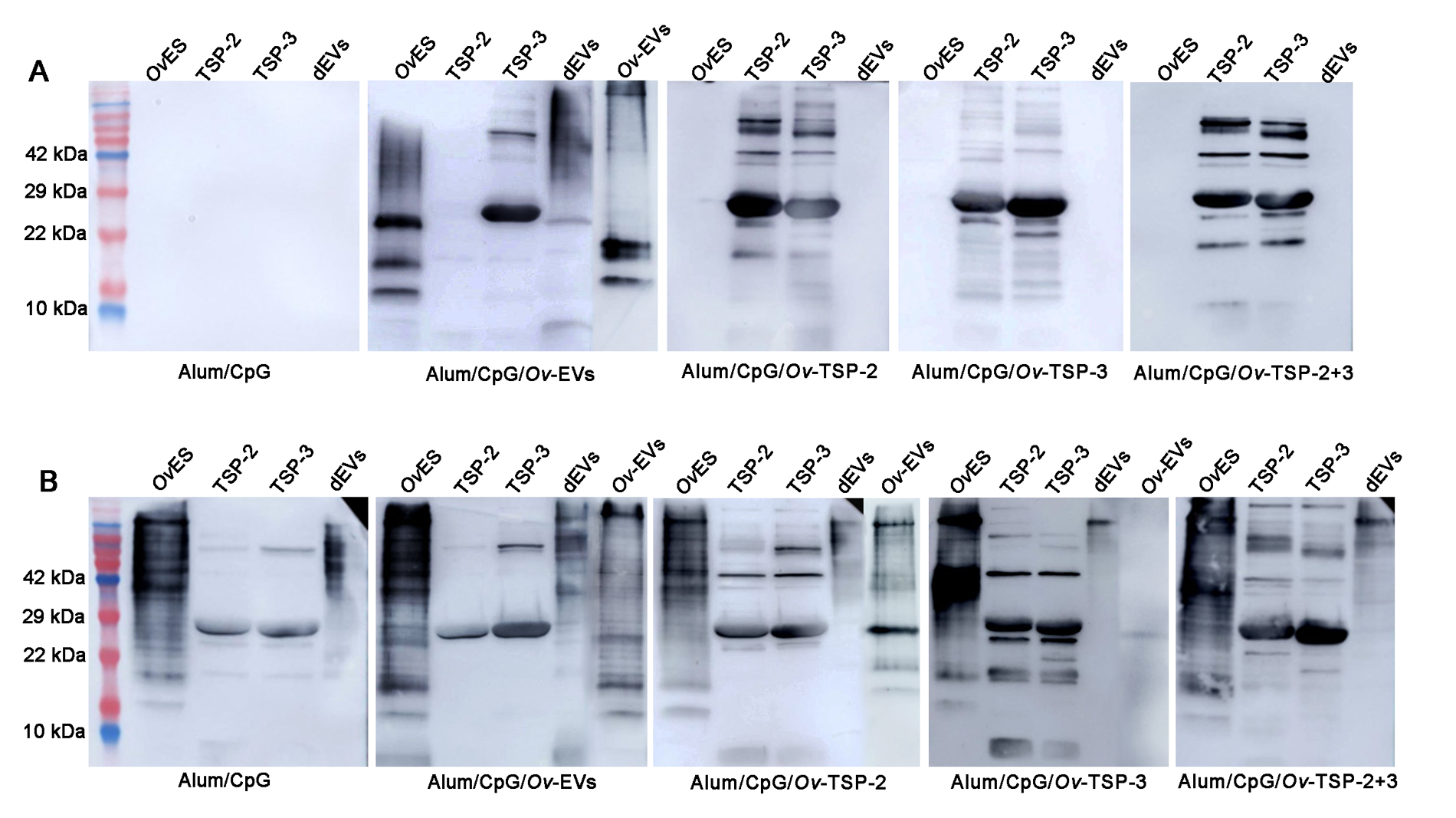

Supplement: S2 Fig — OvES, rOv-TSP-2, rOv-TSP-3, Ov-ES-depleted EVs (dEVs) and Ov-EVs were probed with pre-challenge hamster sera (A) or post-challenge hamster sera from each vaccination group (B). (TIF) [file pntd.0007450.s002.tif]
